# Management of Dislocation of the Shoulder Joint with Ipsilateral Humeral Shaft Fracture: Initial Experience

**DOI:** 10.1111/os.12782

**Published:** 2020-08-19

**Authors:** Fei Lyu, Hui‐xiang Wang, Chun Bi, Shu‐ming Shen, Qiu‐gen Wang, Xiao‐ming Wu

**Affiliations:** ^1^ Department of Orthopaedics Affiliated Hospital of Yangzhou University, Yangzhou University Yangzhou China; ^2^ Department of Orthopaedic Traumatology, Trauma Center Shanghai General Hospital, Shanghai Jiao Tong University School of Medicine Shanghai China

**Keywords:** Case series, Functional outcome, Humeral shaft fracture, Shoulder dislocation, Surgery

## Abstract

**Objective:**

To gain a better understanding of the traumatic mechanism and to develop appropriate treatment for dislocation of the shoulder joint with an ipsilateral humeral shaft fracture.

**Methods:**

This was an observational and descriptive study. Nine patients with traumatic shoulder dislocations associated with ipsilateral humeral shaft fractures who visited the emergency room and received treatment from January 2012 to June 2018 were retrospectively analyzed. CT with three‐dimensional reconstruction was performed to provide precise anatomical information of the fractures. The traumatic event and the type of fracture of the humeral shaft were analyzed to help determine the trauma mechanism. Closed reduction of the dislocation was attempted at once under intravenous anesthesia. One patient died the following day due to unrelated causes. All humeral shaft fractures of the eight patients received internal fixation, and then reduction of the dislocation was performed again if previous attempts failed. The affected limb was immobilized in a sling for 3 weeks postoperatively, and then active and passive movement was encouraged. Patients were evaluated based on clinical and radiographic examinations, shoulder joint range of motion, Constant–Murley score, and subjective shoulder value.

**Results:**

Four cases in the present study could not give a clear description of the traumatic procedure. The other five patients suffered a second strike on their upper arms when they were hurt, with low mobility and high pain in the shoulder region. Seven cases were simple fractures and two were wedge fractures. According to the AO/OTA classification system, four cases were type 12‐A2, three were type 12‐A3, and two were type 12‐B2. Six patients successfully obtained closed manipulative reduction of the shoulder dislocation in the acute stage. The follow‐up time ranged from 18 to 31 months. No deep wound infections were encountered. All fractures healed uneventfully. The union time ranged from 4 to 6 months. At the final follow‐up, shoulder range‐of‐motion values were found to range from 140° to 170° forward flexion, 30° to 40° extension, 40° to 45° adduction, 150° to 170° abduction, 50° to 60° internal rotation, and 50° to 60° external rotation; no recurrent instability of the shoulder joint occurred; the Constant–Murley score was 89.5 ± 3.7 points (range: 84–94 points); the subjective shoulder value was 89.4% ± 6.3% (range: 75%–95%).

**Conclusion:**

Shoulder dislocation most likely occurs first with an axial force or a direct posteroanterior force and a subsequent force results in the shaft fracture. For patients with mid‐distal humerus fractures, closed manipulative reduction of the joint is usually effective. After success of closed reduction, surgery for the humeral shaft fracture is advocated to ensure stability and to make patient nursing convenient. In cases with fractures in the proximal third of the humeral shaft, fixation is suggested beforehand to help reduce the shoulder dislocation.

## Graphical abstract

To describe a treatment strategy for shoulder dislocation associated with a concomitant ipsilateral humeral shaft fracture.

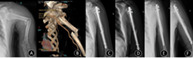

## Introduction

With the largest range of motion among all joints, the glenohumeral joint also has the highest dislocation rate^1^, ^2^. In the United States, it was reported to be 23.9 per 100,000 person‐years^1^. Anterior dislocation is the most common direction of instability following a traumatic event and shows abimodal age distribution. The largest group of individuals who experience shoulder dislocations are young adult men who have sustained high‐energy injuries to the shoulder. The second largest group are older patients who have generally suffered from a much lower level of violence. In addition, shoulder dislocation usually proves to be an isolated event in older patients^3^.

As a shallow articulation, the glenohumeral joint is stabilized by the rotator cuff muscles that attach to the joint capsule, as well as the tendons of the biceps and triceps brachii. The labrum attached to the outer rim of the glenoid fossa provides additional depth and stability, securing the humeral head. Violent external rotation in abduction levers the head of the humerus out of the glenoid socket, avulsing anterior bony and soft tissue structures in the process. A fall onto the outstretched arm, transmitting the force to the glenohumeral joint is a typical mechanism for anterior dislocation.

Acute dislocation is a surgical emergency and demands urgent relocation. Nonoperative management is the most common method of treatment, and there are many techniques for reduction of primary anterior shoulder dislocation. Among them, traction‐based or leverage‐based techniques are more likely to be used and success rates in general range from 60% to 100% regardless of approach^4^. Traction‐based techniques and leverage techniques rely heavily on the continuity of the upper limb, especially the skeletal continuity. However, by reviewing the literature we could see that when shoulder dislocation is complicated with an ipsilateral humeral shaft fracture, there is still no consensus on the management, especially on the reduction sequences of dislocation. Some case reports show a successful closed reduction of the shoulder joint, but some failed^5^, ^6^, ^7^, ^8^, ^9^, ^10^, ^11^, ^12^, ^13^, ^14^, ^15^, ^16^, ^17^, ^18^, ^19^, ^20^, ^21^. The major obstacle in achieving closed reduction is lack of adequate lever arm, which demands surgical intervention to fix the shaft, followed by closed reduction.

The humeral shaft is commonly defined as the segment distal to the surgical neck and proximal to the epicondyles. Fractures of the humeral shaft account for approximately 3% of all long‐bone fractures^22^. The radial nerve arises from the posterior cord of the brachial plexus and runs anterior to the subscapularis muscle to penetrate the triangular interval in conjunction with the deep brachial artery, which may be injured primarily or iatrogenically. For management of isolated humeral shaft fractures, several treatment strategies have been proved to be effective, including functional bracing, open reduction–internal fixation (ORIF), minimally invasive plate osteosynthesis, intramedullary nailing, and external fixation. Each of these modalities has its own advantages and disadvantages. For the present, most surgeons consider nonsurgical treatment for humeral shaft fractures the standard method. However, nonsurgical treatment might be associated with some complications, such as nonunion, malunion, skin abrasion, limited range of motion (ROM), and long‐lasting treatment. In cases in which the humeral shaft fracture also has proximal or distal intraarticular extension and the intraarticular fracture meets operative indications, the humeral shaft fracture is often fixed in the same setting. Furthermore, polytrauma is considered to be a relative indication for humeral shaft fracture. Dislocation of the shoulder joint with an ipsilateral humeral shaft fractures is usually combined with injuries in other sites, such as rib fractures, scapular fractures, and pneumothorax, and treatment would be challenging for most surgeons. Due to the exceedingly low incidence of this combined injury, only a few authors have described their experiences, based on one or two patients.

Gupta *et al*.^16^ fixed the shaft fracture with an external fixator prior to shoulder reduction. Kapila *et al*.^23^ used open reduction with the standard deltopectoral approach to fix the shaft fragment with a long plate first, after which closed reduction of the joint was done. Here we present our experience of a series of nine patients with shoulder dislocation associated with a concomitant ipsilateral humeral shaft fracture, eight of whom underwent surgical treatment. The purpose of this study is as follows: (i) to gain a better understanding of the traumatic mechanism; (ii) to aid in developing appropriate treatment against this combined injury; and (iii) to evaluate the prognosis of patients with shoulder dislocation associated with an ipsilateral humeral shaft fracture.

## Patients and Methods

### *Subjects*

The inclusion criteria for enrolling patients were as follows: (i) patients aged ≥18 years; (ii) patients who visited the emergency room and received treatment between January 2012 and June 2018; (iii) patients with dislocation of the shoulder joint with an ipsilateral humeral shaft fracture according to imageological examination, with times of injury ranging from 0 to 72 h; (iv) the clinical data, including X‐rays and CT with three‐dimensional reconstruction, were complete; and (v) patients who were retrospectively recruited and provided informed consent.

This is an observational and descriptive study. Approval was given by the institutional review board and informed consent was obtained from each patient. According to the patient records from the trauma center of our hospital between January 2012 and June 2018, there were a total of 625 patients with first‐time traumatic humeral shaft fractures. Among them, we identified nine patients (1.44%) with ipsilateral shoulder dislocations. All nine patients were men, with ages ranging from 35 to 60 years. The injury severity score (ISS) in the emergency department ranged from 9 to 32 points. The injury mechanism included a fall in three cases and a traffic accident in six cases. Seven cases were simple fractures and two were wedge fractures. According the AO/OTA classification system, four cases were type 12‐A2, three were type 12‐A3, and two were type 12‐B2. More details are shown in Table 1.

**Table 1:** Demographics and clinical and radiographic findings

Case number	Sex	Age (years)	Affected side	Etiology	ISS	Dislocation	AO classification (fracture characters)
1	Male	51	Left	Traffic accident	29	Anterior	12‐B2 (Mid/3,Varus, Medial Fragment, Greater Tuberosity Fracture)
2	Male	60	Right	Fall	20	Anterior	12‐A3 (Mid‐High/3, Varus)
3	Male	46	Left	Traffic accident	18	Anterior	12‐A2 (Low/3, Varus, Greater Tuberosity Fracture)
4	Male	47	Left	Traffic accident	32	Anterior	12‐A3 (Low/3,Varus, Greater Tuberosity Fracture)
5	Male	50	Left	Fall	9	Anterior	12‐A2 (Mid/3, Varus)
6	Male	35	Right	Traffic accident	22	Anterior	12‐A2 (Mid‐High/3, Varus, Greater Tuberosity Fracture)
7	Male	39	Right	Traffic accident	26	Anterior	12‐B2 (Mid/3, Varus, Medial Fragment)
8	Male	52	Left	Traffic accident	18	Anterior	12‐A3 (Low/3, Varus)
9	Male	58	Left	Fall	14	Anterior	12‐A2 (Low/3, Varus)

ISS, injury severity score.

### *Diagnostic Evaluation*

All nine patients' conditions were evaluated in the emergency department, and immediate consultation with associated specialists was carried out. Upon initial examination, an obvious severe deformity of the humerus was noted in all nine cases. Relevant radiographs including the shoulder and elbow joints showed both humeral shaft fractures and anterior shoulder dislocations. Four cases also had fractures of the greater tuberosity (Table 1). Clinical examination revealed no neurovascular injury.

CT with three‐dimensional reconstruction was performed to provide precise anatomical information of the fractures and help to divide them. Humeral shaft fractures were apparent in the upper third in two cases, the middle third in three cases, and the lower third in four cases. Seven were transverse fractures and the other two were short oblique fractures (Table 1).

### *Treatment Strategy*

An attempt at closed reduction of the shoulder dislocation was made under intravenous sedation on the admission day, which included traction–counter traction by grasping the proximal fragment and pushing the humeral head. Considering the general condition of the patients, the unstable state of the fractured humerus, and the risk of further soft tissue and iatrogenic neurovascular damage, repeated attempts of closed reduction were avoided. A neurovascular exam was performed after the procedure.

The closed reduction, whether successful or not, was followed by temporary coaptation plaster splint. When condition permitted, fixation of humeral fractures was conducted as soon as possible to help reduce the dislocation if the initial manual reduction failed. Meanwhile, humeral shaft fractures in patients whose shoulder dislocation had already been reduced were also fixed with an interlocking intramedullary nail (Trigen PHN, Smith and Nephew) or a metaphysis locking compression plate (LCP, Synthes) according to the radiologic findings. The time between injury to surgery for patients except one who had died ranged from 1 to 12 days.

**Fig 1:**
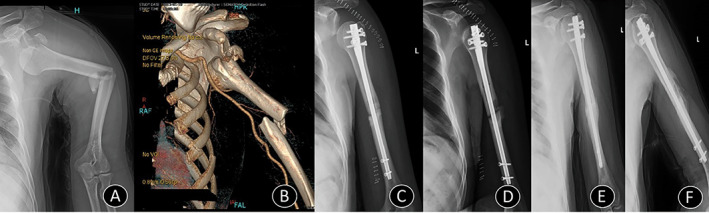
Case 1. (A) A plain radiograph shows dislocation of the shoulder with fractures of the greater tuberosity and humerus shaft. (B) A corresponding three‐dimensional reconstruction of a CT scan. (C) and (D) Immediate postoperative X‐rays showing the fracture fixed with interlocking nail and shoulder reduced, with deltoid palsy. (E) and (F) Six‐month follow‐up X‐ray showing union at the fracture site with reduced shoulder and recovery of deltoid palsy.

**Fig 2:**
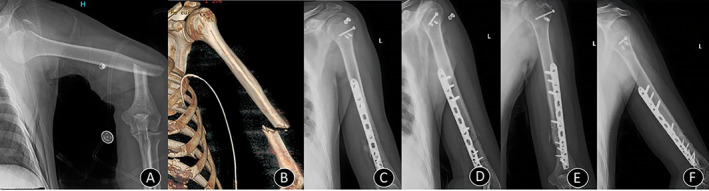
Case 3. (A) A plain radiograph showing dislocation of the shoulder with fractures of the greater tuberosity and humerus shaft. (B) A three‐dimensional reconstruction of a CT scan after reduction of dislocation. (C) and (D) Immediate postoperative X‐rays showing the humerus fracture fixed with locking compression plate and greater tuberosity fracture fixed with cannulated compression screws with rotator cuff tears repaired with suture anchors. (E) and (F) Four‐month follow‐up X‐ray showing union at fracture sites with reduced shoulder.

Greater tuberosity fractures were fixed with proximal interlocking screws when intramedullary nailing was applied (Fig. 1) or cannulated compression screws (4.0 mm, Synthes, Fig. 2) if the displacement exceeded 5 mm in CT scans. MRI of the shoulder was not routinely performed; however, if rotator cuff tears were identified during the operation, they would be repaired with suture anchors or sutured through bone tunnels, and completed with direct suturing (Fastin Anchor, Depuy, Fig. 2).

The affected limb was immobilized in a sling for 3 weeks postoperatively, and then active and passive movement was encouraged.

### *Clinical Assessment*

Patients were evaluated based on clinical and radiographic examination, shoulder joint range of motion, Constant–Murley Score, and subjective shoulder value.

#### *Constant–Murley* *Score*

The Constant–Murley score (CMS) was used to evaluate postoperative recovery of shoulder function in the adult population. The CMS score was 100 points, which consisted of pain (15 points), muscle strength (25 points), functional activity (20 points), and shoulder mobility (40 points). Higher scores indicate better functionality. Objective evaluation indicators included shoulder mobility and muscle strength (65 points), and subjective evaluation indicators included pain and functional activities (35 points).

#### *Subjective Shoulder Value*

The subjective shoulder value (SSV) was defined as a patient's subjective shoulder assessment, expressed as a percentage of an entirely normal shoulder, which would score 100%. It is usually based on a single question that is answered subjectively by the patients. The English formulation of this question is: “What is the overall percent value of your shoulder if a completely normal shoulder represents 100%?”

## Results

### *Reduction of Shoulder Dislocation*

Among the nine patients, closed reduction was performed successfully in six cases. No iatrogenic neurovascular damage was noted after the manual procedure. Although transient postoperative deltoid palsy was noted postoperatively in case 1, full recovery was achieved 6 months after the surgery (Fig. 1).

### *Fracture Union and Complications*

Case 4, with polytrauma, died of multiple organ failure the day after admission. For the remaining eight patients, the follow‐up time ranged from 18 to 31 months. No deep wound infections were encountered. All fractures healed uneventfully. The union time ranged from 4 to 6 months.

### *Shoulder Range of Motion*

**Fig 3:**
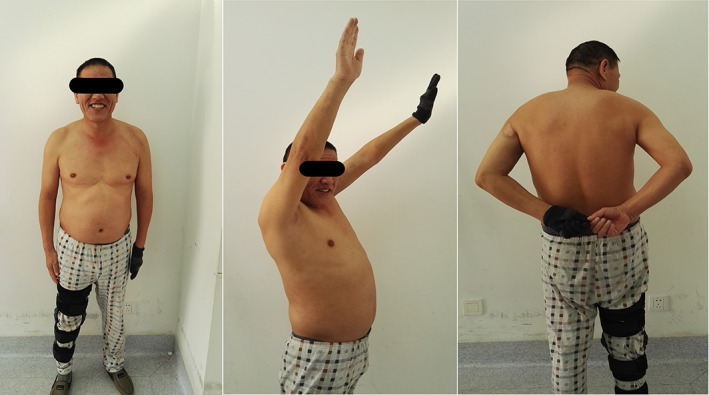
Case 1. Functional results 6 months postoperatively.

At the final follow up, 18 and 31 months after surgery, shoulder range‐of‐motion values were found to range from 140° to 170° forward flexion, 30° to 40° extension, 40° to 45° adduction, 150° to 170° abduction, 50° to 60° internal rotation, and 50° to 60° external rotation (Fig. 3).

### *Constant–Murley* *Score and Subjective Shoulder Value*

The Constant–Murley Score was 89.5 ± 3.7 points (range, 84–94 points); the subjective shoulder value was 89.4% ± 6.3% (range, 75%–95%). No recurrent instability of the shoulder joint occurred at the last follow up (Table 2).

**Table 2:** Treatments and outcomes

Case number	Manual reduction of dislocation	Reduction time (days)	Humerus fracture treatment	Greater tuberosity fracture	Follow up (months)	ROM (°) (F/E/IR/ER/Ab/Ad)	CMS	SSV (%)
1	Failed	12	Intramedullary nailing	Proximal interlocking screws	31	150/35/60/50/160/40	86	90
2	Failed	10	LCP	/	26	160/40/60/60/170/40	90	92
3	Successful	0	LCP	Cannulated screws	28	145/30/55/50/165/45	92	95
4	Successful	0	/	/	/	/	/	/
5	Successful	0	LCP	/	23	170/40/60/60/170/45	94	90
6	Failed	1	Intramedullary nailing	Proximal interlocking screws	24	140/40/55/50/160/40	84	75
7	Successful	0	Intramedullary nailing	/	20	160/30/50/50/150/45	86	88
8	Successful	0	LCP	/	26	170/35/60/60/160/45	92	95
9	Successful	0	LCP	/	18	170/40/60/60/170/45	92	90

Note: Case 4 died the following day after admission. CMS, Constant–Murley score; F/E/IR/ER/Ab/Ad, flexion/extension/internal rotation/external rotation/abduction/adduction; ROM, range of motion; SSV, subjective shoulder value.

## Discussion

### *Epidemiological Characteristic*

The glenohumeral joint is the most commonly dislocated joint in the human body^3^. The majority of patients are young adult men who have sustained high‐energy trauma to the shoulder^3^. Humeral shaft fractures, which occur mainly in the elderly and are often the result of a fall, account for approximately3% of all long‐bone fractures^22^. In our hospital, dislocation of the shoulder joint with an ipsilateral humeral shaft fracture accounted for 1.44% of all humeral shaft fractures. Some orthopaedic surgeons mistake shoulder dislocations for shaft fractures when confronting this rare combined injury^5^, ^6^, ^7^, ^8^, ^9^, ^10^. Therefore, the practice of taking radiographs of the joint above and below a fracture is emphasized. To the best of our knowledge, only 27 cases of shoulder dislocations complicated with ipsilateral humeral shaft fractures have been reported in the literature since 1940. Among them, most were adult men, who suffered from a road traffic accident, which was consistent with the present study (Table 3).

**TABLE 3:** Reported cases of a combination of shoulder dislocation with ipsilateral humeral shaft fracture in adults

Author/date	Age/sex	Etiology	Dislocation	Shaft fracture	Dislocation treatment	Fracture treatment	Follow up/results
Winderman/1940	68 F	Fall	Anterior, GTF	Mid/3	Closed with pin to assist	Splint	3 m/Fair
Milch/1949	27 M	Fall	Anterior	High/3	Closed	Splint	?/Ulnar nerve neuropraxia
Baker/1971	25 M	Operating Machinery	Anterior, GTF	Mid/3	Open	Intramedullary pin	15 m/Fair
John/1978	41 M	Traffic Accident	Posterior (missed)	Mid/3	Open (2 months postinjury)	Splint	10 m/Limitation of motion but back to work
Barquet/1985	23 M	Operating Machinery	Anterior, GTF	Low/3	Closed	Splint	16 m/Good
	42 F	Traffic Accident	Anterior	High/3	Closed	Splint	8 m/Good
Sankaran‐Kutty/1989	28 M	Traffic Accident	Anterior, GTF	Mid/3	Closed after external fixation	External fixator	4 m/Good
Canosa/1994	16 M	Traffic Accident	Anterior	Mid/3	Closed after fracture fixation	Intramedullary pin	12 m/Good
Kontakis/1995	41 M	Traffic Accident	Anterior	Mid/3	Open	Plate	22 m/Good
	45 M	Fall	Anterior	Mid/3	Closed	Splint	12 m/Good
Calderone/1995	27 M	Traffic Accident	Anterior	Mid/3	Closed	Plate	24 m/Good
Davick/1995	29 M	Traffic Accident	Anterior	High/3	Closed	Splint	12 m/Brachial plexopathy
	67 M	Traffic Accident	Anterior	Mid/3	Closed	Intramedullary nailafter reduction of the joint	12 m/Fair
Maffulli/1996	19 M	Traffic Accident	Anterior, GTF	Low/3	Closed after internal fixation	Plate	18 m/Limitation of motion but back to work
Chen/1998	35 M	Traffic Accident	Anterior	Low/3	Closed	Plate	36 m/Good
	28 M	Traffic Accident	Anterior	Low/3	Closed	Plate	12 m/Radial nerve palsy
Micic/2005	18 F	Traffic Accident	Anterior (diagnosed at 45 days post injury)	Mid/3	Open	External fixator	3 y/Good
Sasashige/2006	18 M	Traffic Accident	Anterior	Mid/3	Closed	RetrogradeIntramedullaryNail	11 y/Good
Inan/2008	27 M	Operating Machinery	Anterior, GTF	Mid/3	Closed	Plate	15 m/Good
John/2008	69 F	Fall	Anterior, 3 part	Mid/3	Closed	Splint	2.5 y/Good
Kazakos/2009	33 M	Fall	Anterior	Mid/3	Closed after internal fixation	IntramedullaryNail combined with plate	3 y/Good
Hitesh/2011	20 M	Traffic Accident	Anterior	High/3	Closed with pin to assist	IntramedullaryNail	2 y/Good
Mohammad/2012	15 M	Operating Machinery	Anterior, GTF	Mid/3	Closed	Splint	15y/Good
Kamran/2014	27 M	Traffic Accident	Anterior	High/3	Open	Plate	6 m/Good
Yogendra/2015	30 F	Traffic Accident	Anterior, GTF	Mid/3	Closed after external fixation	External fixator	11 m/Good
Herzberg/2017	84 M	Fall	Anterior, 3 part	High/3	/	Long stem hemiarthroplasty with screws	30 m/Good
Kapila/2018	62 F	Fall	Anterior	High/3	Open	Plate	12 m/Good

GTF, greater tuberosity fracture; IN, intramedullary nail; m, month(s); y, year(s).

### *Traumatic Mechanism*

Dislocation of the shoulder joint with an ipsilateral humeral shaft fracture is commonly a high‐energy injury, and the traumatic mechanism is complex and controversial. Up to now, there has been no clear picture of the sequence of occurrence between a shoulder dislocation and a humeral shaft fracture. Some authors propose that direct transmission of force along the axis of the humerus results in simultaneous dislocation and fracture^7^, ^8^, ^18^, ^19^, while some postulate that the action of force first leads to the dislocation and then subsequent force leads to the fracture^5^, ^11^, ^12^, ^20^. Sankaran‐Kutty and Sadat‐Ali^18^ suggest that a sudden enormous axial loading of the humerus at the flexed elbow with the shoulder in a slightly abducted position is the prerequisite for the simultaneous injuries, through comparison with a similar injury in the lower extremity where a fracture of the femoral shaft was associated with an ipsilateral hip dislocation. However, this explanation is inadequate from a biomechanical point of view^21^ and cannot explain additional greater tuberosity fractures, which are common in high energy shoulder dislocations and appeared in four cases in our study. Bahrs *et al*.^24^ suggest that, in shoulder dislocation, shearing against the glenoid rim is the leading mechanism of greater tuberosity fractures. When the humeral shaft is fractured, the interrupted axial loading can not easily result in shearing or impingement.

Biomechanically, the fracture type is related to the mode of forces acting upon the bone. However, most of the previous reports obtained only X‐rays of rather poor quality and did not classify the fracture types. With the development of CT multislices and 3D reconstruction, the fractures can be classified more easily and precisely, which is one of the advantages of our study. Usually, a transverse fracture is produced by pure bending and an oblique fracture by an uneven bending^25^. Of the nine cases, four were type 12‐A2, three were type 12‐A3, and two were type 12‐B2 according the AO/OTA classification system (Fig. 1), which strongly indicated a direct force on the humeral shaft.

In addition, analysis of how injuries occurred could help to understand the trauma mechanism. Although four patients in the present study were unable to give a clear description of the traumatic event, the other five patients described that when they were hurt, with low mobility and high pain in the shoulder region, their upper arms then suffered a second strike, which might have caused the humeral fracture. Similar descriptions are reported in the published literature. Therefore, we believe that shoulder dislocation occurs first with an axial force or a direct posteroanterior force and then a subsequent force results in the shaft fracture. Generally, the injuries for this group of patients is severe and complicated. In our study, the ISS scores of four cases were greater than 16, and in three cases were greater than 25 (one of them died the following day after admission). A thorough examination is important. Regarding the affected limbs, missed diagnosis of the concomitant shoulder dislocation has been reported on more than one occasion^6^, ^7^, ^9^. Visible deformity and sharp pain of the humerus fracture may mask the symptoms of dislocation. Therefore, the practice of taking radiographs of the joints above and below the fracture is needed. Attention should be paid to distal neurovascular lesions both before and after the reduction of the dislocation.

### *Reduction of Shoulder Dislocation*

Lack of adequate lever arm to conduct the closed reduction of the joint and the unstable fracture may increase the risk of failure and iatrogenic neurovascular damage. Based on this viewpoint, some authors advocate that fixation of humeral shaft fractures should be performed prior to joint reduction^5^, ^8^, ^12^, ^15^, ^16^, ^18^, ^21^. However, in our study, closed manipulative reduction of the shoulder joint succeeded in six patients with mid‐distal humerus fractures but failed in case 1 with interposition of the tendon and cases two and six with proximal humerus fractures. There was no neurovascular lesion after the manual reduction. Therefore, in our experience, closed manipulative reduction of the shoulder joint may achieve a high success rate in patients with mid‐distal humerus fractures. Repeated attempts must be avoided, which strongly suggested that the capsule^21^ or the tendon of the long head of biceps brachii (case 1) might be interposed as an obstacle.

### *Treatment of Humeral Fractures*

When the closed reduction is successful, a variety of treatment methods, including closed reduction with immobilization or open reduction with a plate or a nail, can be used for humeral fractures. Good clinical results have been reported for almost all the modalities. However, the results obtained with different methods cannot be compared due to heterogeneity. Although nonsurgical treatment for humeral shaft fractures has been considered the standard of care^25^, we suggest dislocation of the shoulder joint with an ipsilateral humeral shaft fracture as a relative indication for fracture fixation, because this would provide a firm fixation to allow early commencement of range of motion exercises^10^ and improve the mobility of those patients with polytrauma. Meanwhile, fracture fixation would make the nursing staff convenient in multiple trauma patients. In the present study, all of the five cases with successful closed reduction had undergone subsequent surgeries for the humeral fractures and achieved excellent operative results.

When the fracture occurred in the proximal third of the humeral shaft, considering the lack of adequate lever arm, closed reduction of the joint before fracture fixation seemed difficult (cases two and six). Besides, a patient was once reported to develop nerve damage after a failed attempt at closed reduction^26^. Finally, this type of fracture has a higher nonunion rate than the distal two‐thirds when treated conservatively^27^, ^28^, suggesting that it is better to fix the fracture first. Open reduction and plate fixation is generally the preferred method compared with minimally invasive plating and intramedullary nailing for humeral shaft fractures^22^. However, in the scenario presented above, intramedullary nail fixation is more advantageous^12^. One of the main reasons is that it may jeopardize the stability of osteosynthesis in the case of plate fixation if reduction of dislocation needs to be done after fixation^14^. The advantages of minimally invasive insertion and biomechanically superior constructs also make it more attractive for polytrauma patients. Furthermore, the insertion of the nail is favored by the anteriorly dislocated humeral head because the head is not obstructed by the acromion^12^.

Exceptionally, in case 2, with an upper shaft fracture, minimally invasive plate osteosynthesis *via* the anterior approach was used after the reduction of dislocation had failed. This was because the patient had an ipsilateral fracture of acromial bone and insertion of an intramedullary nail would further aggravate the damage and might contribute to postoperative shoulder pain.

### *Rate of Redislocation*

Recent evidence indicates that the rate of recurrence of primary anterior shoulder dislocation ranges from 19% to 88% at a minimum 2‐year follow up^29^. Male sex and younger age are linked to an increased risk of recurrent instability^29^. To our knowledge, no redislocation occurred in either the present study or the previous case reports. Wasserstein *et al*.^29^ revealed that the concomitant greater tuberosity fracture could significantly decrease the risk of subsequent recurrent dislocation. It was speculated to be attributed to prolonged immobilization, additional activity modifications, and/or reduced mobility secondary to the development of a hemarthrosis^30^. From the view of injury mechanism, the force leading to the fracture of the humeral shaft will inevitably exacerbate the joint injury, thus resulting in a more serious hemarthrosis and soft tissue damage. Subsequent prolonged immobilization and more conservative functional exercise may play a role as well.

### *Limitations*

The study has a few limitations. The number of cases is small due to the scarcity of the combined injury, so an observational and descriptive approach was used. In addition, the severity and complicated circumstances of the injury as well as the lower incidence make it difficult to set a control group to make conclusions regarding the advantages of our method. However, the patients enrolled were injured within a relatively short time span and treated by the same group of surgeons, thus avoiding the impact of doctor‐related factors that may affect treatment decisions and outcomes.

### *Conclusion*

Shoulder dislocation most likely occurs first with an axial force or a direct posteroanterior force, and a subsequent force results in the shaft fracture. For patients with mid‐distal humerus fractures, closed manipulative reduction of the joint is usually effective. After success of closed reduction, surgery for the humeral shaft fracture is advocated to ensure stability and to make patient nursing convenient. In cases with a fracture in the proximal third of the humeral shaft, fixation is suggested beforehand to help reduce the shoulder dislocation.
